# Mechanistic insights into the inhibition of Sec61-dependent co- and post-translational translocation by mycolactone

**DOI:** 10.1242/jcs.182352

**Published:** 2016-04-01

**Authors:** Michael McKenna, Rachel E. Simmonds, Stephen High

**Affiliations:** 1 Faculty of Life Sciences, University of Manchester, Michael Smith Building, Manchester M13 9PT, UK; 2 Department of Microbial Sciences, School of Bioscience and Medicine, Faculty of Health and Medical Sciences, University of Surrey, Guildford GU2 7XH, UK

**Keywords:** Buruli ulcer, Endoplasmic reticulum, *Mycobacterium ulcerans*, Mycolactone, Sec61, Short secretory protein

## Abstract

The virulence factor mycolactone is responsible for the immunosuppression and tissue necrosis that characterise Buruli ulcer, a disease caused by infection with *Mycobacterium ulcerans*. In this study, we confirm that Sec61, the protein-conducting channel that coordinates entry of secretory proteins into the endoplasmic reticulum, is a primary target of mycolactone, and characterise the nature of its inhibitory effect. We conclude that mycolactone constrains the ribosome–nascent-chain–Sec61 complex, consistent with its broad-ranging perturbation of the co-translational translocation of classical secretory proteins. In contrast, the effect of mycolactone on the post-translational ribosome-independent translocation of short secretory proteins through the Sec61 complex is dependent on both signal sequence hydrophobicity and the translocation competence of the mature domain. Changes to protease sensitivity strongly suggest that mycolactone acts by inducing a conformational change in the pore-forming Sec61α subunit. These findings establish that mycolactone inhibits Sec61-mediated protein translocation and highlight differences between the co- and post-translational routes that the Sec61 complex mediates. We propose that mycolactone also provides a useful tool for further delineating the molecular mechanisms of Sec61-dependent protein translocation.

## Abstract (precis)

**Summary:** The Buruli ulcer toxin mycolactone inhibits both the co- and post-translational pathways for protein translocation across the endoplasmic reticulum by perturbing the function of the Sec61 translocon.

## INTRODUCTION

Mycolactone is a polyketide-derived virulence factor produced by *Mycobacterium ulcerans*, the pathogen responsible for the tropical disease Buruli ulcer (George et al., 1999). Buruli ulcer is characterised by chronic and extensive progressively necrotising skin ulcers (Walsh et al., 2011), and histopathology reveals atypical clusters of extracellular bacilli, as well as an absence of infiltrating immune cells (Silva et al., 2009). Mycolactone is responsible for these symptoms, and strains that lack its polyketide-synthase-encoding plasmid produce only short-lived granulomatous infections (Stinear et al., 2004). Mycolactone has been implicated in the under-production of several proteins that are involved in the inflammatory response (Hall et al., 2014; Pahlevan et al., 1999; Simmonds et al., 2009; Torrado et al., 2007), and it is responsible for impaired cell adhesion (Guenin-Macé et al., 2013) as well as a lack of pain reception in patients (Marion et al., 2014). Despite the broad inhibition of protein production observed in Buruli ulcer, mycolactone has no direct negative effect on either transcription or translation of the affected proteins (Boulkroun et al., 2010; Hall et al., 2014; Simmonds et al., 2009). Instead, mycolactone blocks the Sec61-dependent translocation of proteins into the endoplasmic reticulum (ER) leading to their rapid degradation (Hall et al., 2014; Ogbechi et al., 2015), though the precise mechanism by which this occurs is unclear.

Proteins that are synthesised in the cytosol and targeted to the ER include secretory and membrane-embedded proteins, and are often characterised by a hydrophobic stretch of amino acids at or near their N-terminus termed the ‘signal sequence’ (Blobel and Dobberstein, 1975). The majority of these proteins are delivered co-translationally to the ER of mammalian cells (Nyathi et al., 2013). In this pathway, the signal sequence is recognised by the signal recognition particle (SRP) upon emerging from the ribosomal exit tunnel, and the rate of translation is slowed, allowing the substrate to be targeted to the ER as part of a ribosome–nascent-chain complex (RNC) (Mary et al., 2010; Walter and Blobel, 1981a,b; Walter et al., 1981). At the ER, the RNC interacts first with the SRP receptor (Gilmore et al., 1982a,b), and subsequently with the Sec61 translocon (Song et al., 2000), at which point translation continues and protein translocation into the ER lumen occurs.

Some proteins are unable to use the co-translational pathway for entry into the ER and must be delivered post-translationally. These include tail-anchored proteins, which possess a hydrophobic C-terminal targeting sequence that only emerges from the ribosomal exit tunnel after translation has been terminated. Tail-anchored proteins utilise a post-translational pathway that is dependent on TRC40 (also known as ASNA1) to reach the ER, and upon delivery are integrated into the ER membrane in a Sec61-independent manner (Hegde and Keenan, 2011). Another group of proteins capable of using a post-translational route to the ER are the short secretory proteins (SSPs), whose short mature domain means that translation is often terminated before their N-terminal signal sequence has the opportunity to interact co-translationally with SRP (Johnson et al., 2013b). Cytosolic factors, including calmodulin (Shao and Hegde, 2011) and TRC40 (Johnson et al., 2012), have been implicated in promoting SSP delivery to the ER but, in contrast to the integration of tail-anchored proteins, the translocation of SSPs across the ER membrane is dependent on Sec61. Hence, both the small-molecule inhibitor eeyarestatin (Cross et al., 2009; Johnson et al., 2012), and the siRNA-mediated depletion of Sec61α (Lang et al., 2012) perturb the translocation of model SSPs into the ER lumen.

The Sec61 translocon is a heterotrimeric membrane protein complex [comprising Sec61α1 (isoform 1), Sec61β and Sec61γ] that is an essential component for protein translocation into the ER (Görlich and Rapoport, 1993). Based on structural studies of the equivalent archaeal complex (Van den Berg et al., 2004), the Sec61α subunit is proposed to have ten transmembrane domains that form a gated protein-conducting channel across the ER membrane, as well as a ‘lateral gate’ that allows partitioning of hydrophobic domains into the lipid phase of the ER membrane (Martoglio et al., 1995). Recent high-resolution structural studies of the eukaryotic translocon have vastly improved our understanding of Sec61-dependent translocation (Becker et al., 2009; Gogala et al., 2014; Pfeffer et al., 2015; Voorhees et al., 2014; Voorhees and Hegde, 2016), and it is clear that this process is concomitant with conformational changes of the Sec61 complex that constitute channel gating.

The co-translational pathway is highly efficient, with GTP-dependent protein synthesis providing the force necessary for unidirectional translocation through the Sec61 translocon. Although in some instances this process requires the ribosome, Sec61 and SRP receptor only (Görlich and Rapoport, 1993), auxiliary factors, such as the TRAM protein and the TRAP complex, can often enhance translocation in a signal sequence-specific manner (Fons et al., 2003; Lang et al., 2012; Voigt et al., 1996). In contrast, the Sec61-mediated, post-translational translocation of many SSPs, typically less than 120 residues long, is dependent upon Sec62, but shows no requirement for SRP receptor (Lakkaraju et al., 2012). Indeed, a recent study suggests that the Sec61 translocon exists in two mutually exclusive states – a co-translational (SRP-receptor-bound) state and a post-translational (Sec62-bound) state (Jadhav et al., 2015). Likewise, in the absence of ongoing protein synthesis, the directionality of post-translational translocation is provided by ER luminal components, most notably BiP (also known as HSPA5) (Matlack et al., 1999; Schäuble et al., 2012; Tyedmers et al., 2003). In short, although the Sec61 complex can mediate both co- and post-translational translocation into the ER, there is evidence that these two pathways are mechanistically distinct.

In this study, we establish that mycolactone induces a conformational change in Sec61α, the central component of the ER translocon. This change correlates with distinct perturbations of both co- and post-translational translocation pathways into the ER. For example, although we observe efficient and indiscriminate inhibition of co-translational ER translocation, short and unstructured precursors that employ a post-translational route are less prone to mycolactone-mediated inhibition of the Sec61 complex. We conclude that mycolactone induces a wide-ranging, but by no means global, block on ER translocation. Taken together, our findings highlight the complexity of ER translocation and establish mycolactone as an important tool for understanding different modes of Sec61-mediated translocation.

## RESULTS

### Mycolactone efficiently inhibits co-translational translocation of secretory proteins

To assess the ability of mycolactone to inhibit protein translocation into and across the endoplasmic reticulum (ER) membrane, mRNA coding for potential substrates was translated *in vitro* using rabbit reticulocyte lysate (RRL) in the presence of ER-derived canine pancreatic rough microsomes (Hall et al., 2014). Efficient translocation across or insertion into the rough microsomes can be detected by signal sequence cleavage (‘c’ versus ‘nc’) and/or N-glycosylation (‘+1g’ and/or ‘+2g’), depending on the substrate being studied (Fig. S1A). Substrates lacking endogenous glycosylation sites include an additional C-terminal opsin tag termed ‘OPG’ or ‘OPG2’ (one or two consensus N-glycosylation sites, respectively) where indicated. Given that mycolactone does not directly affect translation (Hall et al., 2014), a reduction in the amount of membrane-associated processed substrate provides a reliable readout for an inhibition of ER translocation.

**Fig. 1.**
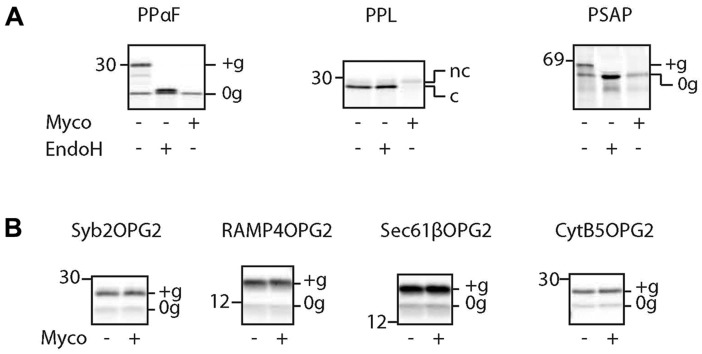
**Mycolactone blocks co-translational translocation into the ER but does not affect tail-anchored protein integration.** Phosphorimage of the indicated *in vitro* synthesised co-translational substrates (A) or tail-anchored proteins (B) in the presence or absence of mycolactone (Myco). Samples were treated with endoglycosidase H (EndoH) where indicated to distinguish glycosylated (+g) from non-glycosylated (0g) bands. nc, signal sequence not cleaved; c, signal sequence cleaved.

As previously described (Hall et al., 2014), the co-translational translocation of yeast prepro-α-factor (PPαF) was effectively blocked by mycolactone (Fig. 1A). Likewise, efficient processing, and hence ER translocation, of the classic secretory protein precursors preprolactin (PPL, also known as PRL) and preprosaposin (PSAP) was seen in the absence but not in the presence of mycolactone (Fig. 1A). In contrast, the membrane integration of four different tail-anchored proteins was unaffected by mycolactone (Fig. 1B). These findings support our proposal that mycolactone targets a key component of the co-translational translocation pathway that is not involved in tail-anchored protein biogenesis (Fig. S1B). Our data also clearly show that mycolactone does not interfere with N-glycosylation within the ER lumen per se, and hence this modification provides a faithful readout for mycolactone-induced inhibition of ER translocation.

### Mycolactone alters the interaction between the RNC complex and the Sec61 translocon

**Fig. 2.**
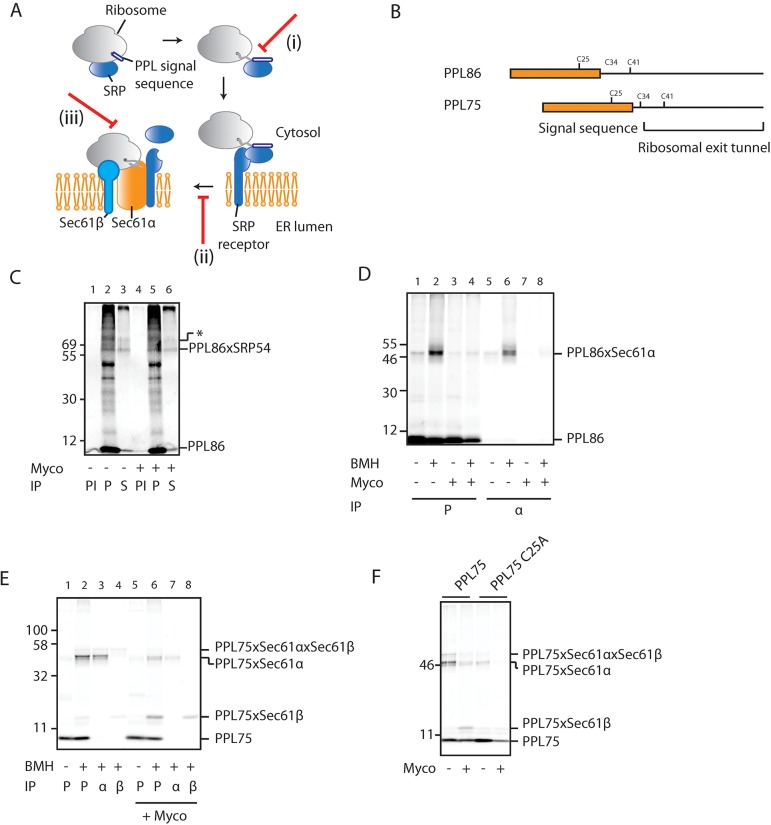
**Mycolactone alters the interaction between RNCs and the Sec61 complex.** (A) Diagram of the co-translational pathway with potential sites of mycolactone inhibition indicated. Scenario (i) – mycolactone might interfere with the ability of the signal sequence to engage SRP. Scenario (ii) – mycolactone might prevent the transfer of RNCs from the SRP receptor to the Sec61 complex, perhaps by preventing a productive interaction between these two components. Scenario (iii) – mycolactone might alter the interaction of RNCs with the Sec61 complex. (B) Schematic of PPL truncations used for crosslinking analysis in this study, with cysteine residues highlighted. (C) Phosphorimage of DSS-crosslinked (crosslinking indicated by ‘x’) PPL86 that had been *in vitro* translated with or without mycolactone (Myco) and in the absence of rough microsomes. Samples were immunoprecipitated (IP) using antisera raised against either PPL (P) or SRP54 (S). Control samples were mock immunoprecipitated with pre-immune rabbit serum (PI). In addition to an adduct with SRP54 (PPL86×SRP54), a higher molecular mass species is also observed (*). We speculate that this most likely represents an adduct that contains an additional component such as SRP19. Phosphorimages of BMH-crosslinked crosslinked PPL86 (D) and PPL75 (E) that had been *in vitro* translated with or without mycolactone and in the presence of rough microsomes. Samples were immunoprecipitated after carbonate extraction using antisera raised against either PPL (P), Sec61α (‘α’) or Sec61β (‘β’). (F) Phosphorimage of BMH-crosslinked PPL75 and PPL75 C25A that had been *in vitro* translated with or without mycolactone.

Previous studies have identified several different stages of the co-translational translocation pathway at which inhibitors can act, including SRP binding, RNC transfer to the ER, and polypeptide translocation through the Sec61 complex (Kalies and Römisch, 2015). We therefore set out to define the point at which mycolactone perturbs co-translational translocation (Fig. 2A). To this end, mRNA coding for a truncated version of PPL that lacks a stop codon (PPL86, Fig. 2B) was translated *in vitro* to generate a stable RNC complex that results in a trapped translocation intermediate (Gilmore et al., 1991). The nearest neighbours of these artificial translocation intermediates can then be investigated by employing protein crosslinking techniques (Cross et al., 2009; MacKinnon et al., 2014).

To address the possibility that mycolactone affects the ability of nascent PPL86 chains to interact with SRP (Fig. 2A, scenario i), RNCs were generated in the absence of rough microsomes to enable SRP binding, and the samples were treated with the bifunctional amine-reactive crosslinking reagent disuccinimidyl suberate (DSS). Immunoprecipitation using antisera raised against the 54-kDa subunit of SRP (SRP54) confirmed the identity of discreet crosslinking between it and nascent PPL86, which persisted when the PPL86-RNCs were generated in the presence of mycolactone (Fig. 2C, lane 3 versus lane 6). The persistence of the PPL86×SRP54 adduct in the presence of mycolactone was also confirmed using the alternative crosslinking reagent SMCC (Fig. S2A). We therefore conclude that mycolactone does not perturb the binding of SRP to nascent PPL86 chains.

When PPL86-RNCs were generated in the presence of rough microsomes, the nascent chain formed a strong crosslink with Sec61α when treated with the thiol-selective crosslinking reagent bismaleimidohexane (BMH) (Fig. 2D, lane 6). Strikingly, this adduct almost completely disappeared in the presence of mycolactone (Fig. 2D, lane 8). To establish whether this loss of crosslinking to Sec61α represents a failure of the RNC to reach the Sec61 complex (Fig. 2A, scenario ii), a shorter nascent PPL chain (PPL75, Fig. 2B) that potentially reflects an earlier stage of the ER translocation process (Laird and High, 1997) was analysed. PPL75 generates adducts to both Sec61α and Sec61β (Fig. 2E), and whereas the adduct with Sec61α was clearly diminished in the presence of mycolactone (Fig. 2E, lane 3 versus lane 7), crosslinking to Sec61β was noticeably enhanced (Fig. 2E, lane 4 versus lane 8). The amino acid residue primarily responsible for these adducts was identified as Cys25 within the PPL signal sequence (Fig. 2B,F; Fig. S2B). Furthermore, mycolactone showed no ability to prevent the re-binding of ribosomes to ER-derived microsomes that had been first stripped of ribosomes by EDTA treatment and high-salt washing (Fig. S2D). Given that ribosome binding at ER-derived membranes is primarily due to interaction with the Sec61 complex (Kalies et al., 1994), we conclude that mycolactone does not prevent the RNC from reaching the Sec61 complex (Fig. 2A, scenario ii), but rather alters the nature of their interaction (Fig. 2A, scenario iii).

### Mycolactone partially inhibits the post-translational ER translocation of short secretory proteins

**Fig. 3.**
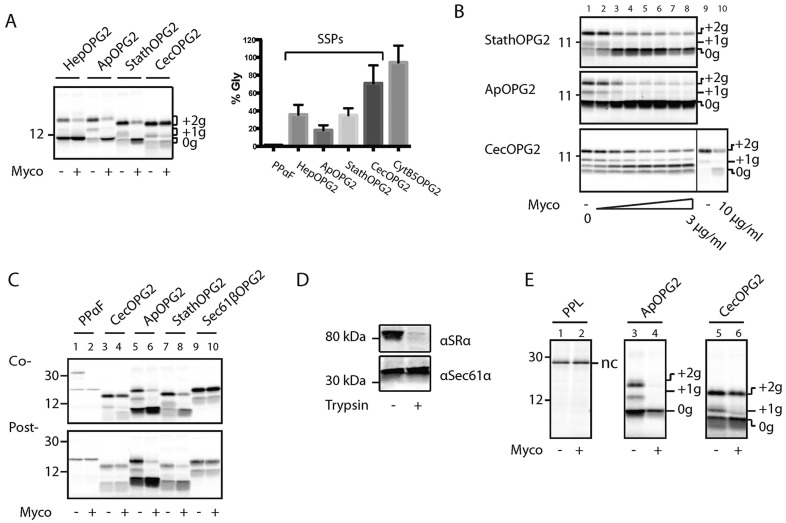
**Mycolactone partially inhibits post-translational SSP translocation into the ER.** (A) Various SSPs containing C-terminal OPG2 tags [hepcidin (HepOPG2, 102 residues), apelin (ApOPG2, 95 residues), statherin (StathOPG2, 82 residues) and cecropin (CecOPG2, 82 residues)] were *in vitro* translated with or without mycolactone (left panel), and their ability to translocate was assessed by dividing the amount of doubly glycosylated material (+2g) in the presence of mycolactone (Myco) by the amount in the absence of mycolactone (right panel). Results are mean±s.d. (for CecOPG2 *n*=9; for StathOPG2 and CytB5OPG2, *n*=6; for all other substrates, *n*=8). 0g, non-glycosylated; +1g, singly glycosylated. (B) StathOPG2, ApOPG2 and CecOPG2 were *in vitro* translated in the presence of increasing concentrations of mycolactone. Final concentrations (µg/ml) of mycolactone from left to right are: 0, 0.2, 0.5, 1, 1.5, 2, 2.5 and 3. CecOPG2 was also tested with 10 µg/ml mycolactone (lane 10) or an equivalent volume of DMSO only (lane 9). Between 0 and 3 µg/ml mycolactone, CecOPG2 was recovered using native immunoprecipitation as opposed to membrane recovery by high-speed centrifugation in order to demonstrate that the decrease in glycosylated material was not due to an inhibition of total material. (C) Translocation of SSPs into rough microsomes in the presence of mycolactone was tested in either a co-translational (Co-, top panel) or a post-translational (Post-, bottom panel) *in vitro* translocation system. (D) Western blot of salt-washed rough microsomes (KRMs) that had been incubated with or without 1 µg/ml trypsin on ice for 1 h using antisera raised against either SRα or Sec61α (C-term). (E) The indicated substrates were *in vitro* translated with or without mycolactone in the presence of trypsinised KRMs. nc, signal sequence not cleaved.

Having found that mycolactone blocks the co-translational translocation of secretory polypeptides through the Sec61 complex, we next wished to test its effect on short secretory protein (SSP) translocation. We investigated four SSPs, all of which include a C-terminal OPG2 reporter: hepcidin (HepOPG2), apelin (ApOPG2), statherin (StathOPG2) and cecropin (CecOPG2). Like tail-anchored proteins, SSPs can enter the ER lumen through a post-translational and ribosome-independent mechanism (Johnson et al., 2013b), but unlike tail-anchored proteins, they do so through the Sec61 translocon (Cross et al., 2009; Johnson et al., 2012; Lang et al., 2012). Unlike the classical secretory protein substrates, which were efficiently blocked by mycolactone (Fig. 1A), and tail-anchored membrane proteins, which were unaffected (Fig. 1B), SSPs showed an intermediate level of ER translocation when synthesised in the presence of mycolactone (Fig. 3A). SSP translocation persisted even at concentrations threefold higher than those capable of fully blocking longer secretory proteins (Fig. 3B, lane 8). Furthermore, with CecOPG2, translocation was still clearly evident when mycolactone was present at a level tenfold greater than that used to inhibit co-translational translocation (Fig. 3B, lane 9 versus lane 10), ruling out the likelihood of any simple differences in dose dependence for different classes of substrate. In fact, the amount of ER-translocated material changed little beyond mycolactone concentrations of 0.5 µg/ml for StathOPG2, 1 µg/ml for ApOPG2 and 1.5 µg/ml for CecOPG2 (Fig. 3B, lanes 3, 4 and 5, respectively), suggesting that these SSPs possess an inherent partial resistance to mycolactone-dependent inhibition of Sec61-mediated translocation.

Although SSPs can enter the ER lumen post-translationally, it is possible that a proportion of newly synthesised precursors might access the co-translational SRP-dependent pathway when synthesised in the presence of ER microsomes (cf. Lakkaraju et al., 2012). In order to establish whether mycolactone is selectively inhibiting such a co-translational pool, SSP translocation was analysed using an experimental protocol that is strictly post-translational, given that ER microsomes were added after translation had been terminated and any residual nascent chains released from their ribosome. Although PPαF translocation was fully blocked by mycolactone in the co-translational system (Fig. 3C, ‘Co-’, lanes 1 and 2), we observed no translocation in the post-translational system even in the absence of mycolactone (Fig. 3C, ‘Post-’, lanes 1 and 2), confirming PPαF as an obligate co-translational substrate in our cell-free system. In contrast, the translocation of SSPs was apparent in this post-translational system and this process was inhibited by mycolactone to a similar extent as observed when protein synthesis was carried out in the presence of ER microsomes (Fig. 3C, lanes 3–8 compare ‘Co-’ and ‘Post-’). The SRP-dependent pathway can also be selectively disabled by limited trypsinisation, which degrades the α-subunit of the SRP receptor α (SRα, also known as SRPR) while leaving the pore-forming α-subunit of the Sec61 complex largely intact (Abell et al., 2004; Fig. 3D). Hence, following trypsinisation, obligate co-translational substrates such as PPL cannot be translocated and processed by the signal peptidase (Fig. 3E, lanes 1 and 2). Nevertheless, efficient SRP-independent translocation of both ApOPG2 and CecOPG2 was observed following this trypsinisation (Fig. 3E, lanes 3 and 5), and the inhibitory effect of mycolactone was similar to that previously observed (Fig. 3E, lanes 4 and 6, compare with Fig. 3B). We, therefore, conclude that mycolactone also inhibits Sec61-dependent translocation when it occurs in a strictly post-translational manner, but the extent of this inhibition appears to be substrate specific.

### Inhibition of SSP translocation by mycolactone is dependent on both signal sequence identity and mature domain length

**Fig. 4.**
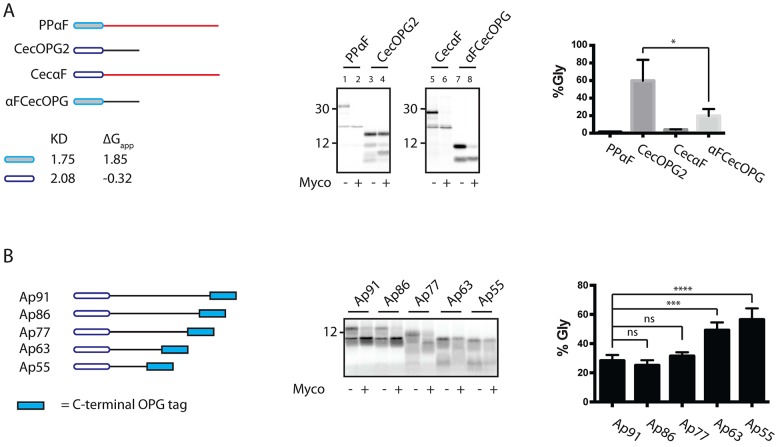
**Inhibition of SSPs by mycolactone is dependent on signal sequence identity and mature domain length.** (A) Chimeras of PPαF and CecOPG2 were generated by swapping of their respective signal sequences (left panel) and their translocation into rough microsomes in the presence of mycolactone (Myco) was assessed as described above (middle and right panels). Results are mean±s.d. (*n*=3). The net hydrophobicity (Kyte-Doolittle scale, KD) of the full PPαF and CecOPG2 signal sequences were estimated by averaging the individual values obtained using http://web.expasy.org/protscale/ (window size of 9). Alternatively, the Δ*G*_app_ for the same regions was calculated using http://dgpred.cbr.su.se/ (Hessa et al., 2007). It should be noted that more hydrophobic signal sequences are denoted by more positive KD values and more negative Δ*G*_app_ values. (B) A series of apelin truncations, including C-terminal OPG tags, were generated (left panel) and their translocation into rough microsomes in the presence of mycolactone was assessed (middle and right panels). Results are mean±s.d. (*n*=4). The mycolactone sensitivity of the longest apelin-derived construct (Ap91) was found to be significantly different to both Ap63 and Ap55. ***P≤0.001; ****P≤0.0001; ns, not significant (one-way ANOVA).

Having observed that the post-translational translocation of different SSPs into the ER lumen was blocked by mycolactone to different extents, we next wished to investigate what determined this specificity. Since some inhibitors of Sec61 are highly signal sequence-dependent (Kalies and Römisch, 2015), we first addressed the potential role of the signal sequence in determining mycolactone sensitivity. To do this, αFCecOPG and CecαF, previously characterised chimeric proteins containing the signal sequences from PPαF (co-translational and highly mycolactone sensitive) and CecOPG2 (post-translational and highly mycolactone resistant), respectively, were analysed together with their parental proteins (Fig. 4A; Johnson et al., 2013a). Although parental CecOPG2 is particularly refractive to mycolactone inhibition (Fig. 3A,B), replacing its endogenous signal sequence with that of PPαF generated a post-translational αFCecOPG chimera that was noticeably more sensitive to mycolactone (Fig. 4A, lanes 3, 4, 7 and 8). However, although the signal sequence of CecOPG2 is more hydrophobic than that of PPαF (see Fig. 4A and accompanying legend), replacing the normal PPαF signal sequence with that of CecOPG2 had little effect, and translocation of the resulting CecαF chimera was also efficiently inhibited by mycolactone (Fig. 4A, lanes 1, 2, 5 and 6). Taken together, these data suggest that signal sequence identity plays some role in determining the mycolactone sensitivity of post-translationally translocated SSPs, but does not make a major contribution to the mycolactone-induced inhibition of co-translationally translocated substrates.

In addition to signal sequence identity, the sensitivity of SSP translocation to mycolactone appeared to show some relationship to the length of the mature domain of the protein (Fig. 3A). For example, ApOPG2 (95 residues long including the signal sequence) was inhibited more effectively by mycolactone than the shorter CecOPG2 (82 residues including the signal sequence). To test this potential relationship further, a series of apelin truncations that all retained the same C-terminal OPG reporter were generated. When the length of these apelin-derived polypeptides was reduced from 91 to 63 or 55 residues, mycolactone was noticeably less effective at inhibiting their ER translocation, resulting in a higher proportion of N-glycosylation following mycolactone treatment (Fig. 4B). We therefore conclude that the size of the mature domain can contribute to the mycolactone sensitivity of SSPs.

### Trapping a post-translational translocation intermediate at the Sec61 translocon

**Fig. 5.**
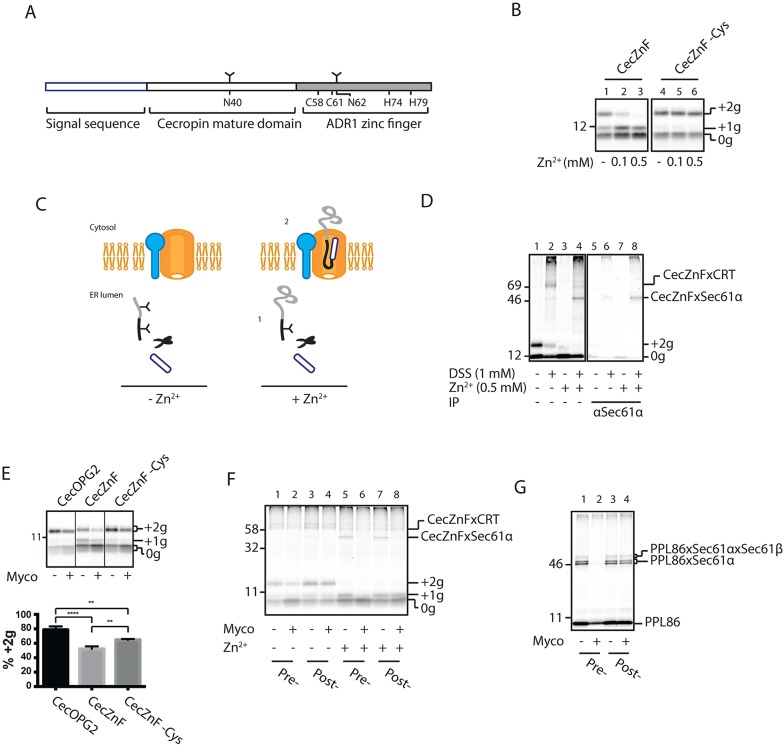
**Mycolactone affects co- and post-translationally trapped intermediates at the Sec61 translocon differently.** (A) Schematic of CecZnF indicating N-glycosylation sites (Y symbols), as well as cysteine and histidine residues involved in coordinating the zinc ion in the C-terminal ADR1 zinc finger domain. (B) Post-translational translocation of CecZnF and CecZnF −Cys (a folding deficient mutant) into rough microsomes with increasing concentrations of exogenous zinc ions (Zn^2+^). 0g, non-glycosylated ; +1g, singly glycosylated; +2g, doubly glycosylated. (C) Cartoon depicting the two postulated populations of CecZnF in the presence of exogenous Zn^2+^ (0.5 mM): (1) fully translocated but only singly glycosylated and (2) non-glycosylated and trapped at the Sec61 translocon. (D) DSS crosslinking analysis of CecZnF in the presence or absence of exogenous Zn^2+^ (0.5 mM). Samples were analysed by SDS-PAGE either following isolation of rough microsomes by ultracentrifugation (left panel) or by immunoprecipitation (IP) using antisera raised against Sec61α (C-terminus) (right panel). (E) Post-translational translocation of CecOPG2, CecZnF and CecZnF–Cys was assessed with only endogenous Zn^2+^ ions present in the presence or absence of mycolactone (Myco). Results are mean±s.d. (*n*=3). (F) DSS crosslinking analysis of CecZnF with or without additional Zn^2+^ ions and with mycolactone added either before addition of rough microsomes (Pre-) or after addition of rough microsomes (Post-). (G) As for F, but BMH crosslinking was performed on PPL86.

Although artificially generated RNCs have been extensively used to probe the environment of translocating polypeptides through the co-translational route (Gilmore et al., 1991), this approach is not well-suited to study post-translational translocation. We therefore sought to artificially trap a translocating SSP by incorporating the 29-residue ADR1 zinc finger at the C-terminus of a truncated form of cecropin (herein denoted ‘CecZnF’) such that this region of the polypeptide can form a stable, folded conformation in the presence of zinc ions (Conti et al., 2014). Furthermore, the version of CecZnF that we created included two consensus sites for N-glycosylation (N40 and N62) to provide a reporter for ER translocation (Fig. 5A).

In the absence of added zinc ions, CecZnF is capable of efficient post-translational translocation into ER-derived microsomes, as indicated by N-glycosylation of the polypeptide (Fig. 5B, lane 1). Increasing the concentration of exogenous zinc ions caused the fully glycosylated form of CecZnF to disappear (Fig. 5B, lanes 1–3, see ‘+2g’), and resulted in a concomitant increase in the non-glycosylated form (Fig. 5B, lanes 1–3, see ‘0g’). Addition of zinc ions led to no such change for a mutant form of CecZnF that lacks Cys58 and Cys61 and which therefore cannot co-ordinate zinc ions (hereafter denoted CecZnF −Cys) (Fig. 5B, lanes 4–6). We also noted that addition of zinc ions led to an increase in the amount of singly glycosylated CecZnF (Fig. 5B, lanes 1–3, see ‘+1g’), which we confirmed was due to modification of residue N40 (Fig. S3A). We therefore postulated that binding of zinc ions leads to two distinct populations of CecZnF (Fig. 5C): (1) a singly glycosylated, yet fully translocated population, where N62 is occluded from the oligosaccharyltransferase due to folding of the zinc finger (McGinnes and Morrison, 1997; Schulz et al., 2009); and (2) a non-glycosylated population that is trapped by the Sec61 translocon.

In order to test this hypothesis, we used chemical crosslinking to probe the local environment of CecZnF in the presence and absence of exogenous zinc ions. This approach revealed the appearance of an adduct between CecZnF and Sec61α, together with a reduction in crosslinking between CecZnF and the ER lumenal chaperone calreticulin (CRT, also known as CALR; Fig. 5D; Fig. S3B). Furthermore, upon EndoH treatment, no size shift was observed for the Sec61α adduct (Fig. S3C), confirming that its CecZnF component is not glycosylated, but is instead a bona fide post-translational trapped intermediate. The faint appearance of the CecZnF and Sec61α adduct observed in the absence of additional zinc ions (Fig. 5D, lanes 2 and 6) is most likely due to folding induced by endogenous zinc ions (Conti et al., 2014). Like other SSPs (see Figs 3A and 4A), CecZnF displays only a partial sensitivity to mycolactone, with over 50% of the substrate still being translocated in the presence of the compound (Fig. 5E). Furthermore, CecZnF is slightly more sensitive to mycolactone than its CecZnF −Cys control (Fig. 5E), suggesting that the folding and/or conformation of the mature domain can influence the sensitivity of SSPs to mycolactone.

Despite only partially inhibiting CecZnF translocation, mycolactone strongly diminished the DSS-mediated adduct formed between CecZnF and Sec61α in the presence of zinc ions (Fig. 5F, lane 5 versus lane 6). This observation suggests that mycolactone does not preclude access of the substrate to the translocon pore, but changes the architecture of the translocon in such a way that it prevents the formation of this amine-dependent crosslinking product. Interestingly, mycolactone could disrupt the ability of trapped CecZnF chains to crosslink Sec61α even when it was added after the ER-targeting step had occurred and trapped intermediates had already accumulated (Fig. 5F, lane 7 versus lane 8). In contrast, mycolactone was unable to reverse the juxtaposition of nascent PPL86 chains that had already docked at the Sec61 complex as part of a pre-existing RNC translocation intermediate (Fig. 5G, lane 3 versus lane 4). Therefore, our observations with mycolactone highlight differences between co- and post-translationally delivered secretory proteins both in their initial engagement with the Sec61 complex and subsequent translocation into the ER lumen.

### Mycolactone alters the crosslinking profile and protease-sensitivity of Sec61α

Taken together, the data outlined above support a model where mycolactone induces a conformational change at the ER translocon and/or its associated components that results in defective protein translocation through the Sec61 complex. Having ruled out any effect on ribosome binding (Fig. S2D), we employed crosslinking to probe the local environment of four translocon and translocon-associated components: Sec61α, Sec61β, Sec62 and TRAM (also known as TRAM1), in the presence and absence of mycolactone (Fig. S4A–D). Treatment of rough microsomes with mycolactone resulted in a modest qualitative reduction in the intensity of DSS-dependent adducts containing Sec61α (Fig. S4A, lanes 5 and 6, asterisk) and Sec61β (Fig. S4B, lanes 5 and 6, asterisk). It remains to be confirmed that these two ∼47-kDa products represent a single adduct containing both Sec61α and Sec61β (Kalies et al., 1998). Nevertheless, these data are consistent with a subtle conformational change at the Sec61 translocon following mycolactone treatment, as further suggested by the enhancement of a faint ∼75 kDa BMH-dependent adduct of Sec61β that is observed after mycolactone treatment of rough microsomes (Fig. S4B, lanes 3 and 4, double asterisk).

**Fig. 6.**
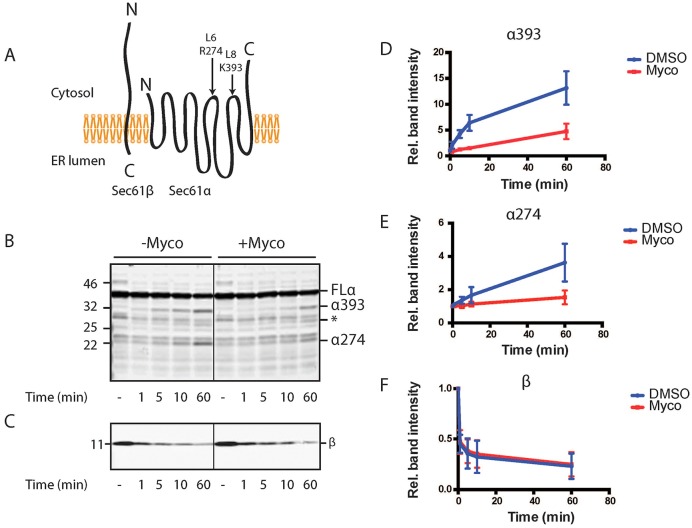
**Mycolactone induces a conformational change in the Sec61α subunit of the ER translocon.** (A) Schematic showing Sec61α and Sec61β topologies in the ER membrane with trypsin cleavage sites in Sec61α loops L6 and L8 indicated. Western blotting of trypsinised KRMs was performed with an antiserum raised against either Sec61α (N-terminus) (B) or Sec61β (C) in the presence or absence of mycolactone (Myco). Degradation products corresponding to cleavage at Sec61α loops L8 (α393) and L6 (α274) are indicated, as well as an unidentified degradation product at ∼27 kDa (*). Full-length Sec61β is also indicated (β). FLα, full-length Sec61α. (D–F) Relative band intensity of α393, α274 and β over the 60-min trypsinisation. Results are mean±s.d. (*n*=3).

As an alternative approach to study mycolactone-induced conformational changes of key translocon components, limited proteolysis was performed using high-salt-washed rough microsomes that had been pre-incubated with either mycolactone or a vehicle control. Trypsinisation of control membranes and blotting for an N-terminal epitope of Sec61α revealed two previously characterised digestion products that arise from cleavage at cytosolic loops L6 and L8 of this polytopic protein (Fig. 6A; see Song et al., 2000). Pre-treatment of membranes with mycolactone resulted in a kinetic delay in the appearance of these proteolytic fragments (Fig. 6B,D,E), indicating that Sec61α had become more resistant to trypsin cleavage at these sites. In contrast, Sec61β showed no difference in sensitivity to trypsin following mycolactone treatment (Fig. 6C,F), thus ruling out the possibility that mycolactone affects trypsin activity per se. Hence, mycolactone treatment alters the protease sensitivity of Sec61α, the central pore-forming component of the ER translocon (Van den Berg et al., 2004).

## DISCUSSION

In this study, we characterise the inhibitory mechanism of the polyketide-derived virulence factor mycolactone, and confirm that its principal target is the Sec61 translocon of the ER membrane. Furthermore, we observe clear differences in the effects of mycolactone on co- and post-translational translocation through the Sec61 translocon. Mycolactone efficiently inhibits co-translational translocation of polypeptides into the ER, whereas post-translationally translocated SSPs typically show only a partial inhibition in the presence of mycolactone. The extent to which SSP translocation is inhibited by mycolactone can apparently be influenced by signal sequence hydrophobicity, as well as the length and folding propensity of the mature domain. In both cases, these effects on protein translocation are likely brought about by a mycolactone-induced conformational change in the pore-forming Sec61α subunit, which we speculate might stabilise a closed conformation of the Sec61 complex (see below). These findings highlight previously unappreciated differences between Sec61-mediated co- and post-translational translocation, and provide further molecular insights into the pathology of Buruli ulcer.

### Mycolactone inhibits co-translational translocation through Sec61

The previously characterised small-molecule inhibitor eeyarestatin I (ESI) has been proposed to inhibit co-translational Sec61-dependent translocation into the ER by preventing the transfer of RNCs from the SRP receptor to Sec61 (Cross et al., 2009), and we find that adducts between a trapped PPL75 nascent chain and both Sec61α and Sec61β are clearly diminished in the presence of ESI (Fig. S2C). However, it is worth noting that whereas ESI treatment completely inhibited crosslinking of Sec61α to a trapped membrane protein intermediate (Cross et al., 2009), it only partially inhibits its crosslinking to PPL75 (Fig. S2C). The difference between these two precursors suggests that the effects of ESI on ER translocon might be more complex than previously envisaged (Cross et al., 2009). In contrast to the effects of ESI, although mycolactone treatment also results in a substantial loss of crosslinking to Sec61α, crosslinking between nascent PPL75 and Sec61β is actually enhanced (Fig. 2E). This finding suggests that rather than preventing the delivery of co-translational substrates to the ER translocon per se, mycolactone changes the nature of the Sec61–RNC interaction at a post-targeting step.

Enhanced crosslinking of nascent polypeptides to Sec61β has been observed with the cyclodepsipeptides CAM741 and cotransin, which selectively perturb ER translocation in a signal-sequence-specific manner (Garrison et al., 2005; Besemer et al., 2005; Harant et al., 2007). In contrast, the mycolactone-dependent inhibition of co-translational substrates appears to be unaffected by the signal sequence identity of the precursors in this study (Figs 1A and 4A), more closely resembling the inhibitory effects of cotransin CT09 (Maifeld et al., 2011). This suggests that mycolactone can inhibit the co-translational translocation of a wide range of substrates, consistent with the near complete loss of glycosylated and secreted proteins observed when mammalian cells are treated with the compound (Hall et al., 2014). Furthermore, although the effects of cyclodepsipeptides on the ER translocation of bona fide SSPs are poorly characterised (Johnson et al., 2013a), the inhibitory effect of mycolactone that we observe on this class of substrate is detailed below.

Although we observe that mycolactone alters the interaction between RNCs and Sec61α, it appears less capable of doing so once the targeting of PPL86-RNCs to the Sec61 complex has already taken place (Fig. 5G). Previous studies have suggested that PPL86-RNCs form a tight interaction with the Sec61 complex that precludes access of cytosolic factors to the nascent chain (Connolly et al., 1989; Hegde and Lingappa, 1996). We therefore speculate that RNC docking at the Sec61 complex might either obstruct the target site of mycolactone, or that, once formed, the stability of this ribosome-bound complex is such that it prevents conformational changes in Sec61α that mycolactone otherwise induces. In support of this, we find that the local environment of an SSP translocation intermediate that is trapped at the Sec61 complex in the absence of a ribosome can be perturbed upon mycolactone treatment (Fig. 5F). However, we cannot rule out the possibility that our trapped SSP translocation intermediate is capable of cycling on and off the translocon, and that mycolactone prevents its proper interaction with Sec61α by acting when our substrate is not bound to the Sec61 complex. It is worth noting that the mycolactone resistance of the RNC docked at the Sec61 complex has been observed using an artificial *in vitro* translocation intermediate. In a physiological setting, the rapid recycling of ribosomes on and off the ER translocon (Jan et al., 2014) would therefore afford no long-term resistance of co-translational substrates to mycolactone.

### Mycolactone has different effects on Sec61-mediated co- and post-translational translocation

Mycolactone inhibits SSP translocation into the ER to a lesser extent than for co-translationally translocated substrates (Fig. 3A versus 1A). Moreover, this partial inhibition persists in a strictly post-translational and ribosome-independent *in vitro* system (Fig. 3C–E), demonstrating that the effect of mycolactone on ER translocation cannot be explained solely by a disruption of the RNC–Sec61 interaction. Rather, mycolactone treatment alters the Sec61 translocon in such a way that limits its role in both co- and post-translational translocation. Interestingly, the production of several chemokines that bear the hallmarks of SSPs is affected in cell culture models (Coutanceau et al., 2007; Hall et al., 2014). We speculate that the properties of these chemokines are akin to the more sensitive SSPs, such as apelin, that we have defined using an *in vitro* system. Alternatively, in a cellular context, mycolactone treatment might simply inhibit the renewal of key cellular components that depend upon the co-translational pathway, including Sec61α (Knight and High, 1998).

Unlike co-translational substrates, the extent of SSP inhibition by mycolactone shows some dependence on the identity of the signal sequence, and we speculate that this might be due to differences in hydrophobicity (see Johnson et al., 2013a). Hence, when the endogenous signal sequence of CecOPG2 is replaced with the less hydrophobic signal from PPαF, the resulting chimera is more sensitive to mycolactone (Fig. 4A). Additionally, the ER translocation of SSPs can be made less sensitive to mycolactone by truncating the mature domain (Fig. 4B) or reducing its propensity to fold prior to translocation (Fig. 5E). It has been shown that signal sequence hydrophobicity must be sufficient to induce translocon gating (Jungnickel and Rapoport, 1995; Trueman et al., 2012), and that point mutations and small-molecule inhibitors can alter this ‘hydrophobicity threshold’ by stabilising either ‘open’ or ‘closed’ conformations of the Sec61 translocon (Junne et al., 2007; MacKinnon et al., 2014; Voorhees and Hegde, 2016). A recent study has identified decatransin as a molecule capable of inhibiting co- and post-translational translocation into the ER, and found this compound to be much less effective when the closed conformation of Sec61 was destabilised by introducing point mutations into the so-called plug domain (Junne et al., 2015). Similar mutations conferred partial resistance to the translocation inhibitor CT8, as did increasing the hydrophobicity of the apolar region responsible for inducing channel gating (MacKinnon et al., 2014). Our observations are therefore consistent with a model where mycolactone stabilises a closed conformation of the Sec61 translocon that, in the case of post-translational translocation, requires a more hydrophobic signal sequence to induce translocon opening, and which is less permissive to the post-translational translocation of mature domains that are long or stably folded. One intriguing possibility is that these different small-molecule inhibitors of the ER translocon, including mycolactone, all bind to a similar region of the Sec61α subunit (MacKinnon et al., 2014, 2007).

The stabilisation of a closed Sec61 conformation by mycolactone is also consistent with our observation that co-translationally translocated substrates are prevented from entering the ER, yet, unlike SSPs, the translocation of these substrates is not enhanced by increasing signal sequence hydrophobicity (Fig. 4A). At present we can only speculate as to the molecular basis for this difference. It could be that a mycolactone-dependent stabilisation of a closed Sec61 conformation can indeed be overcome by increasingly hydrophobic signal sequences present on co-translational substrates, but the translocation of their large mature domains is still precluded, in contrast to the shorter and less structured SSPs (Figs 3A, 4B and 5E). Alternatively, mycolactone might critically interfere with ribosome-dependent priming of the Sec61 complex (Pfeffer et al., 2015; Voorhees and Hegde, 2016). Hence, although mycolactone has no effect on the binding of ribosomes to ER membranes (Fig. S2D), we observe discrete changes in the architecture of the RNC–Sec61 interaction as evidenced by crosslinking of the nascent chain to Sec61 subunits (Fig. 2E). Additionally, the cytosolic regions of Sec61α that show altered trypsin sensitivity upon mycolactone treatment (Fig. 6B,D,E) overlap with domains that are implicated in ribosome binding (Cheng et al., 2005; Voorhees et al., 2014). On this basis, we favour a model where mycolactone perturbs an interaction between the ribosome and the Sec61 complex that is necessary for co-translational translocation to progress (Becker et al., 2009; Cheng et al., 2005; Gogala et al., 2014; Pfeffer et al., 2015; Voorhees and Hegde, 2016), and thereby efficiently inhibits this pathway irrespective of the precise signal sequence carried by a precursor protein. In summary, mycolactone inhibits both co- and post-translational translocation through the Sec61 translocon, and provides a promising tool for further delineating the complexities of protein translocation across the ER membrane.

## MATERIALS AND METHODS

Synthetic mycolactone A/B was a gift from Yoshito Kishi, Harvard University, MA (Song et al., 2002). Unless otherwise stated, all standard laboratory reagents were obtained from Merck or Sigma.

### DNA constructs

HepOPG2 and CecZnF were obtained from Genscript and subcloned into pcDNA5 (Invitrogen). All other short secretory protein and tail-anchored protein constructs were as previously described (Johnson et al., 2012; Rabu et al., 2008). CecOPG2 and PPαF chimeras were prepared as described previously (Johnson et al., 2013a). PPαF was from Jeffrey Brodsky (University of Pittsburgh, Pittsburgh, PA). PSAP was obtained from Origene. PPL was as described previously (High et al., 1993). cDNAs were generated by PCR and transcribed with T7 polymerase (Promega).

### Antibodies

The mouse monoclonal antibody recognising the opsin tag (Adamus et al., 1991) and the rabbit antiserum against Sec61α (N-terminus) (Laird and High, 1997) were as described previously. Rabbit antisera against: SRP54, Sec61β and SRα were gifts from Bernhard Dobberstein (University of Heidelberg, Heidelberg, Germany); Sec61α (C-terminus) and Sec62 from Richard Zimmermann (University of Saarland, Homburg, Germany); and PPL from Sharon Tooze (Francis Crick Institute, London, UK). A rabbit antiserum recognising an internal peptide of the human 25-kDa subunit of the signal peptidase complex (SPCS2) was custom made by Eurogentec. Anti-calreticulin was purchased from Affinity Bioreagents (catalogue number, PA3-900). Anti-RPL19 was purchased from Santa Cruz Biotechnology (catalogue number sc-100830). Antibody dilutions are given in the relevant section.

### *In vitro* translation and translocation assays

Translation reactions (25 µl) were carried out using nuclease-treated rabbit reticulocyte lysate (Promega). Translations were performed in the presence of EasyTag EXPRESS^35^S Protein Labelling Mix containing [^35^S]methionine (Perkin Elmer) (0.769 MBq; 43.5 TBq/mmol). Amino acids minus methionine (Promega) were added to 30 µM. 1 µg of *in vitro* transcribed RNA was then added. For co-translational reactions, 10% (v/v) nuclease-treated rough microsomes [optical density at 280 nm (OD_280_)=44/ml] were added and the samples were incubated for 30 min at 30°C. For post-translational reactions, the sample was incubated for 15 min at 30°C in the absence of rough microsomes. Puromycin was added to 0.5 mM following the translation and incubated at 30°C for 5 min to ensure effective release of the polypeptide from the ribosome. 2 µl of rough microsomes (OD_280_=44/ml) were added and the sample was incubated for a further 20 min at 30°C. Mycolactone in DMSO was first diluted to 25 µg/ml using 0.5% (w/v) BSA in nuclease-free water before addition to the translation mixture to give a final concentration of 1 µg/ml. Control was an equivalent volume of 10% (v/v) DMSO in 0.5% (w/v) BSA. For the co-translational system, mycolactone was present during translation. For the post-translational system, mycolactone was added after puromycin treatment, but before rough microsome addition. To look at folding of the CecZnF zinc finger, ZnCl_2_ solution in 6 mM HCl was added to a final concentration of 0.1 or 0.5 mM after puromycin treatment but before rough microsome addition, and incubated at 30°C for 3 min.

### Membrane recovery and visualisation

Membranes were recovered by centrifugation through an 80-µl high-salt cushion [0.75 M sucrose, 0.5 M KOAc, 5 mM Mg(OAc)_2_, 50 mM Hepes-KOH, pH 7.9] at 100,000 ***g*** for 10 min at 4°C in a TLA100 rotor (Beckmann). The membrane pellet was resuspended in 20 µl low-salt buffer [100 mM sucrose, 100 mM KOAc, 5 mM Mg(OAc)_2_, 50 mM Hepes-KOH pH 7.9, 1 mM DTT] and treated with 250 µg/ml RNase A at 37°C for 10 min. Where indicated, samples were also treated with endoglycosidase H (EndoH) (New England Biolabs) as described by the supplier. The resulting samples were analysed by SDS-PAGE and phosphorimaging using a Typhoon FLA-7000 (GE Healthcare). Images were then processed using Adobe Photoshop and Adobe Illustrator. Data were quantified using Aida (Raytek) and statistical analysis (one-way ANOVA) was performed using GraphPad (Prism). The exact sample size (*n*) for each experimental group is provided in the appropriate figure legends. In each case, *n* was defined by the number of times the substrate was tested in the same experimental system and so represents technical replicates.

### Crosslinking and immunoprecipitation

For crosslinking PPL86 to cytosolic components, PPL86-RNCs were generated by carrying out *in vitro* translation for 15 min in the absence of rough microsomes, as described above. The RNC pellet was isolated by centrifuging 160,000 ***g*** for 20 min at 4°C, before being resuspended in low-salt buffer. DSS (in DMSO stock) was added to a final concentration of 1 mM (control was an equivalent volume of DMSO) and was incubated at 30°C for 10 min before being quenched with 50 mM glycine. Denaturing immunoprecipitation was then carried out by adding SDS to 1% (v/v) and heating at 70°C for 10 min. Nine volumes of Triton immunoprecipitation buffer (10 mM Tris-HCl pH 7.5, 140 mM NaCl, 1 mM EDTA, 1% Triton X-100, 5 mM PMSF, 1 mM methionine) were added, and the appropriate antiserum was added at 1:200. Samples were incubated for 15 h at 4°C with constant agitation. Protein-A–Sepharose beads (Genscript) were added to 10% (v/v) and samples were incubated at 4°C for a further 2 h. Protein-A–Sepharose beads were then recovered by centrifuging at 13,000 ***g*** for 1 min and washed with Triton immunoprecipitation buffer before being heated at 70°C for 10 min in SDS sample buffer. For crosslinking to translocon components, after membranes were recovered and resuspended in low-salt buffer as described above, the appropriate chemical cross-linker was added to 1 mM and incubated at 30°C for 10 min. The reaction was quenched by adding 20 mM β-mercaptoethanol for BMH-treated samples, or 50 mM glycine for DSS-treated samples. For PPL truncations, samples were also carbonate-extracted by adding 150 µl of 0.1 M Na_2_CO_3_ (pH 11.3), incubating for 15 min on ice, and recovering the membrane fraction by centrifuging at 100,000 ***g*** for 10 min at 4°C. Samples were then either analysed directly by SDS-PAGE, or were first immunoprecipitated under denaturing conditions as described above. Native immunoprecipitation of CecOPG2 during mycolactone titration (see Fig. 3B) was performed as above, with the exception that Triton immunoprecipitation buffer was added to the total translation reaction without rough microsome recovery through a high-salt cushion, and no SDS was added.

### Microsome trypsinisation and western blotting

Salt-washed rough microsomes (denoted KRMs) were prepared as described previously (Walter and Blobel, 1983) and mycolactone was added to 2.5 µg/ml. Control was an equivalent volume of 10% (v/v) DMSO in 0.5% (w/v) BSA. KRMs were then treated with 1 µg/ml trypsin on ice for 1 h, with further protease activity being inhibited by the addition of 5 mM PMSF and incubating on ice for 10 min. To degrade Sec61α, KRMs were subjected to a second round of high-salt washing before incubating with 1 µg/ml trypsin on ice. 5 mM PMSF was added at the indicated time points and the samples were incubated on ice for a further 10 min. Samples were then separated by SDS-PAGE and were analysed by using western blotting as described previously (Johnson et al., 2012). For blotting, anti-Sec61α (N-terminus) antibodies were used at a dilution of 1:1000 and anti-Sec61β at 1:3000. Blots were quantified using Image Studio (LI-COR Biosciences).

### Microsome crosslinking and western blotting

To perform crosslinking of translocon subunits and associated components, as seen in Fig. S4, rough microsomes were incubated with either 2.5 µg/ml mycolactone or an equivalent volume of DMSO in 0.5% (w/v) BSA for 5 min at 30°C. Either BMH or DSS was added at 100 µM final concentration and incubated at 30°C for 10 min. The reactions were quenched by adding 20 mM β-mercaptoethanol for BMH-treated samples, or 50 mM glycine for DSS-treated samples. Samples were then carbonate-extracted as described above and were analysed by performing SDS-PAGE and western blotting as described previously (Johnson et al., 2012). For blotting, all primary antibodies were used at a dilution of 1:1000 apart from anti-Sec61β, which was used at 1:3000.

### Ribosome-binding assay

Ribosomes were isolated from rabbit reticulocyte lysate (Promega). 400 µl of lysate was centrifuged at 13,000 ***g*** for 20 min at 4°C to remove debris. The supernatant was then applied onto a cushion of 0.5 M sucrose in binding buffer [20 mM HEPES (pH 7.6), 10 mM NaCl, 5 mM Mg(OAc)_2_, 150 mM KOAc, 1 mM DTT] and centrifuged at 200,000 ***g*** for 30 min at 4°C. The pellet was resuspended in 400 µl of 10% (v/v) glycerol in binding buffer. Ribosomes were again centrifuged at 13,000 ***g*** and 19 µl of the supernatant was added to 2 µl EDTA-treated and high-salt-washed rough microsomes [denoted EKRMs, prepared as described previously (Jadhav et al., 2015)]. The concentration of the EKRMs was equivalent to rough microsomes of OD_280_=44/ml, as determined by blotting for SPC25. Samples were incubated for 10 min at 30°C with 1 µg/ml mycolactone or an equivalent volume of 10% (v/v) DMSO in 0.5% (w/v) BSA. Control samples were incubated with either 5 mM EDTA or rough microsome buffer in place of EKRMs. Membranes were then recovered by centrifuging at 13,000 ***g*** for 10 min at 4°C before being analysed by using SDS-PAGE and western blotting as described previously (Johnson et al., 2012). For blotting, anti-SPC25 was used at 1:2000 and anti-RPL19 at 1:500. The relative amount of bound ribosomes was calculated by dividing the amount of ribosomes present (determined by blotting for RPL19) by the amount of EKRMs (determined by blotting for SPC25).
